# Alterations of Growth Performance, Blood Parameters, and Antioxidant Function of Brown Adipose Tissue in Mice Exposed to Cold

**DOI:** 10.3390/antiox15040476

**Published:** 2026-04-11

**Authors:** Xuekai Zhang, Xiao Jin, Zhipeng Han, Min Jiang, Binlin Shi

**Affiliations:** College of Animal Science, Inner Mongolia Agricultural University, Hohhot 010018, China

**Keywords:** cold exposure, growth, blood parameters, brown adipose tissue antioxidant capacity

## Abstract

Cold exposure is an unavoidable stressor in cold regions, leading to growth retardation, oxidative damage, and endocrine disruption. This study investigated changes in blood parameters and antioxidant function in the brown adipose tissue (BAT) of mice exposed to cold. Sixteen naturally mated female mice (aged 70 days) were selected and divided into a control group (CON, n = 8, 25 ± 1 °C) and a cold exposure group (CE, n = 8, 4 ± 1 °C). Each pregnant female gave birth to approximately 12 pups, and the litter (dams and pups co-housed) served as the independent experimental unit, with both euthanized for sampling when the pups reached 20 days of age. Results showed that cold exposure increased ADFI and ADG but decreased the feed conversion rate (FCR) in lactating mice. It also decreased platelet count (PLT) and mean corpuscular hemoglobin concentration (MCHC), elevated lactate dehydrogenase (LDH) activity, and decreased TG and non-esterified fatty acid (NEFA) levels. Hormonal changes included increased adrenocorticotropic hormone (ACTH), apelin 12 (AP12), INS, NE, decreased cortisol (COR), LEP, and thyroid-stimulating hormone (TSH). In pups, cold exposure inhibited growth, reduced PLT, plateletcrit (PCT), red blood cells (RBC), and hemoglobin (HGB), altered lipid profiles, and induced hormonal shifts. Notably, cold exposure enhanced the BAT antioxidant capacity in pups, increasing the total antioxidant capacity (T-AOC) and antioxidant enzyme activities, as supported by gene expression. These findings suggest that, despite growth suppression, mice maintain homeostasis by modulating blood parameters and enhancing BAT antioxidant function to mitigate cold-induced damage.

## 1. Introduction

Stress is defined as the totality of non-specific responses exhibited by an organism when confronted with stressors [1]. Cold exposure, as a common environmental stressor, profoundly affects physiological functions and the metabolic status in animals. It disrupts homeostatic regulation, triggers oxidative stress, and induces lipid peroxidation, protein oxidation, and nucleic acid damage [2,3]. Prolonged cold exposure may result in significant tissue damage or contribute to the development of cell degeneration-related diseases. As an adaptive response, cold stress redirects energy from growth, lactation, and reproduction toward heat production [4]. It limits feed conversion efficiency, thereby exerting negative effects on animal growth even when feed intake is increased. Under such conditions, the energy metabolism shifts from primarily supporting productive processes to prioritizing the maintenance of body temperature. When animals are exposed to cold environments for extended periods, the secretion of thyroid hormones, total cholesterol (TC), adrenocorticotropic hormone (ACTH), and other hormones in serum increases, which in turn modulates corresponding physiological and metabolic activities [5,6]. In addition, prolonged cold exposure enhances sympathetic–adrenal system activity and induces systemic metabolic adjustments, all of which serve as protective mechanisms to preserve thermal homeostasis [7]. This adaptive process promotes the generation of reactive oxygen species (ROS). Although low levels of ROS may exert immunomodulatory effects, excessive thermogenesis leads to ROS overproduction. When ROS accumulation surpasses the capacity of antioxidant defense systems, oxidative stress ensues, resulting in tissue damage and inflammatory responses [8]. Uncontrolled release of free radicals and their derivatives can damage nucleic acids, enzymes, and biological membranes, ultimately leading to pathological consequences [9]. For instance, cold exposure has been shown to significantly increase hydrogen peroxide (H_2_O_2_) levels in rats while concurrently decreasing total antioxidant capacity (T-AOC) and antioxidant enzyme activity, thereby directly contributing to oxidative damage [10,11]. Similarly, under acute or chronic cold stress conditions, the malondialdehyde (MDA) content in chicken heart tissue significantly increases, while the activity of peroxidase decreases significantly [12]. Collectively, these findings indicate that cold stress not only disrupts normal physiological functions but also exacerbates oxidative stress and antioxidant imbalance, thereby promoting tissue damage and the development of age-related diseases.

In young rodents, BAT plays a dual role in thermoregulation and the regulation of energy metabolism balance [13,14]. BAT is rich in mitochondria that contain uncoupling protein 1 (UCP1), which facilitates proton backflow independently of ATP synthesis, thereby converting chemical energy into non-shivering thermogenesis to maintain body temperature during cold exposure [15]. Beyond its thermogenic function, BAT also exhibits antioxidant properties that help counteract cold-induced oxidative damage, thus providing protective effects for the organism [16]. Oxidative stress is a key contributor to age-related BAT dysfunction, and antioxidant interventions have been shown to ameliorate such damage [17,18]. During cold exposure, the body maintains thermal stability by activating BAT thermogenesis, a process that is tightly regulated by stress-related hormones, including the thyroid hormone (TH), adrenocorticotropic hormone (ACTH), epinephrine (EP), and norepinephrine (NE) [19]. In particular, norepinephrine activates non-shivering thermogenesis in BAT [20]. These hormones directly or indirectly modulate BAT activity, forming a complex physiological network that coordinates thermogenesis and antioxidant defense in response to cold stress.

Pregnancy represents a critical period for fetal development and exerts a direct influence on offspring health and postnatal performance. Cold exposure during pregnancy can lead to adverse outcomes, including impaired fetal development, low birth weight, and postnatal growth suppression [21]. Notably, maternal cold exposure, particularly during late gestation, is recognized as a key environmental factor that affects offspring development [22]. It not only compromises maternal health but also exerts profound effects on the growth, development, and physiological functions of the offspring [23].

Although previous studies have separately investigated the effects of cold exposure on growth performance, hematological parameters, or BAT function in adult animals, few have systematically examined these interconnected aspects in both lactating mice and their offspring within a unified experimental framework. Furthermore, evaluating the relationship between growth and endocrine changes induced by chronic cold exposure during gestation and lactation and the antioxidant capacity of BAT is of great significance for the protection of animal welfare in winter. Therefore, the present study aimed to comprehensively evaluate the effects of cold exposure on growth performance, blood biochemical and hematological parameters, and BAT antioxidant function in mice, with a particular emphasis on age-dependent responses in pups.

## 2. Materials and Methods

### 2.1. Animals and Experiment Design

The experiment was conducted in accordance with the national standard “Guide-lines for Ethical Review of Animal Welfare” (GB/T 35892-2018) [24] and the guidelines and regulations of Inner Mongolia Agricultural University at the mouse experimental base in Hohhot, China. The study was approved by the Institutional Ethics Committee of the Inner Mongolia Agricultural University (No: NND2023107). A total of 16 healthy, naturally mated pregnant mice (70 days of age, body weight: 48.85 ± 1.71 g) were included in this study. Based on comparable body weight, the animals were randomly assigned to two treatment groups (n = 8), with each pregnant mouse giving birth to approximately 12 pups. Mice in the control group (CON) were housed at a temperature of 25 ± 1 °C, while those in the cold exposure group (CE) were maintained at 4 ± 1 °C. The experimental intervention commenced on gestational day 10 and continued for 30 consecutive days. All animals were housed under a relative humidity of 50–60% and a 12 h light/dark cycle, with ad libitum access to a standard maintenance diet (formulated in accordance with GB 13078-2017 and GB 14924.2-2001 [25,26]; detailed composition and nutritional levels are presented in Table 1) and tap water. Offsprings were nursed naturally by their dams. For offspring-related analyses, the litter was regarded as the independent experimental unit, and mean values of individual pups within each litter were calculated for statistical comparisons to avoid pseudo-replication.

On postnatal days 1, 10, and 20, pups were anesthetized by inhalation of 5% isoflurane. Interscapular brown adipose tissue (iBAT) was rapidly dissected, immediately snap-frozen in liquid nitrogen, and stored at −80 °C until subsequent analysis. After tissue collection, pups were euthanized by exposure to diethyl ether. On postnatal day 20, blood samples were collected from the orbital sinus of both dams and pups under anesthesia for the determination of blood biomarkers.

### 2.2. Measurement of Growth Performance

During the experiment, the body weight of all experimental mice was measured daily using an electronic scale (DT-10KA, Changshu Jinyang Weighing Instruments Co., Ltd., Suzhou, China). The initial body weight (IBW) and final body weight (FBW) were recorded. The leftover feed and water were measured and recorded daily to calculate ADG, ADFI, feed conversion ratio (FCR), and average water intake (ADWI).

### 2.3. Blood Collection and Analysis

On the 20th day after birth, both the dams and pups were anesthetized with ether. Blood samples were collected from the retro-orbital sinus and placed in both heparinized and non-heparinized tubes. After sampling, all animals were euthanized. The blood routine was determined using a microhematology method with an automatic blood cell analyzer (PROKAN PE-6800, Jinan Prokan Electronic Instrument Co., Ltd., Jinan, China). The non-heparinized blood was centrifuged at 3000× *g* for 15 min and the supernatant was aspirated and stored at −80 °C for subsequent analysis. These assays were used to measure blood cell counts, biochemical parameters, and hormone levels. Biochemical and hormonal parameters were determined with mice-specific ELISA kits (Ruixin Biological Technology Co., Ltd., Quanzhou, China), according to the manufacturer’s instructions.

### 2.4. Determination of Antioxidant Markers in Brown Adipose Tissue

In pups of different postnatal ages, the iBAT were homogenized into a 10% suspension in saline. The contents of antioxidant indicators including catalase (CAT), total superoxide dismutase (T-SOD), glutathione peroxidase (GSH-Px), total antioxidant capacity (T-AOC), and malondialdehyde (MDA) in BAT were measured using commercial kits according to the instructions of the manufacturer (Nanjing Jiancheng Institute of Bioengineering, Nanjing, China).

### 2.5. Total RNA Extraction and Quantitative RT-PCR Analysis

Total RNA was extracted from BAT using Trizol^TM^ Reagent (TaKaRa Biotechnology Co., Ltd., Dalian, China) following the manufacturer’s recommendations. The quality and quantity of the isolated RNA were determined as described previously [27]. Subsequently, total mRNA was treated with DNase I (TaKaRa Biotechnology Co., Ltd., Dalian, China) to remove genomic DNA contamination, then reverse transcribed into cDNA using the PrimeScript RT™ Master Mix kit (TaKaRa) according to the manufacturer’s protocol on a LifeECO system (Bori Technology Co., Ltd., Hangzhou, China).

Quantitative real-time PCR (qRT-PCR) was performed using TB^®^ Premix Ex Taq™ Kit (TaKaRa Biotechnology Co., Ltd., Dalian, China). Each sample was run in duplicate in 20 μL reaction mixture and performed with cycling conditions as described previously. The LightCycler^®^ 96 Real-Time PCR Design and Analysis System (ROCHE Ltd., Basel, Switzerland) was used for qPCR assays. The qPCR protocol involves the following steps: pre-denaturation at 95 °C for 30 s, PCR amplification stage with 95 °C for 5 s, followed by 60 °C for 34 s, and melting curve analysis with 95 °C for 15 s, 60 °C for 1 min, and finally 95 °C for 15 s. The number of cycles ranged from 35 to 45. The relative alteration in gene expression was performed by 2^−ΔΔCt^. The primer sequences are provided in Table A1.

### 2.6. Statistical Analysis

All data were initially compiled using Microsoft Excel 2021 (Microsoft Corporation, Redmond, WA, USA). Statistical analyses were performed using SAS software (version 9.2, SAS Institute Inc., Cary, NC, USA), and graphs were generated using GraphPad Prism 8 (GraphPad Software, San Diego, CA, USA). For growth performance measured over time, a repeated-measures ANOVA was conducted using the MIXED procedure, with treatment as the between-subjects factor and time as the within-subjects factor. For offspring data, the litter was considered the experimental unit; a linear mixed model was applied, with treatment and age as fixed effects and dam as a random effect to account for the nested structure of pups within litters. For single-time-point measurements (blood parameters), independent samples *t*-tests were used. For two-factor analyses involving treatment and age (antioxidant function), the interaction between factors was evaluated within the mixed-model framework. Multiple comparisons between treatments were performed using Duncan’s multiple range test (DMRT). Prior to statistical analysis, the assumption of normality for all variables was assessed using the UNIVARIATE procedure in SAS. Variables that deviated from a normal distribution were log-transformed to satisfy the assumptions of parametric tests. Post hoc comparisons were performed using Tukey–Kramer adjustment. Data are presented as means ± SEM, with *p* < 0.05 considered statistically significant and *p* < 0.10 considered a tendency.

## 3. Results

### 3.1. Growth Performance

**Figure 1:**
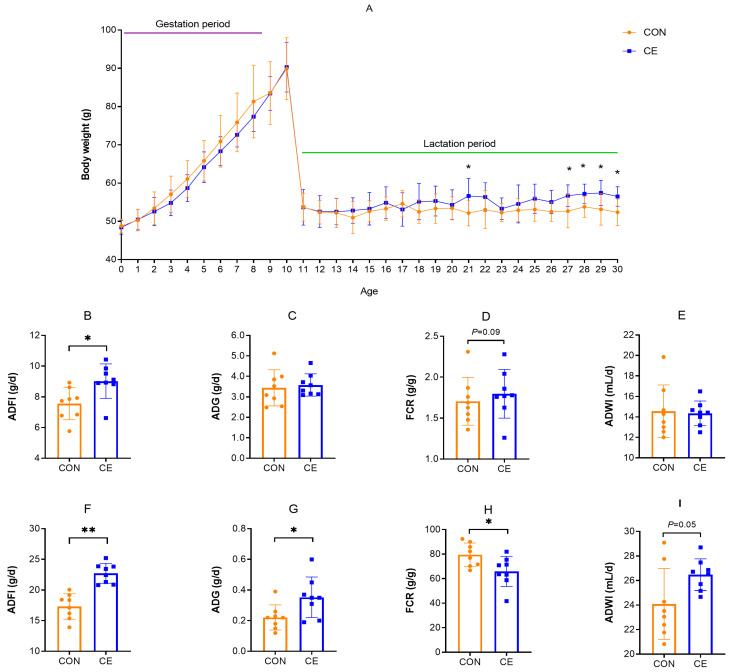
Effect of cold exposure on growth performance of female mice. (**A**) Effect of cold exposure on body weight of female mice. (**B**–**E**) Growth performance of pregnant female mice. (**F**–**I**) Growth performance of lactating female mice. ADFI (average daily food intake), ADG (average daily weight gain), FCR (feed conversion ratio), and ADWI (average daily water intake). Note: * indicates *p* < 0.05, ** indicates *p* < 0.01.

Figure 1 shows the effect of cold exposure on the growth performance in female mice. As shown in Figure 1A, the body weight of pregnant females increased significantly with age, whereas lactating females did not show significant changes in body weight. In pregnant female mice, ADFI was significantly higher in the CE group than in the CON group (*p* < 0.05), and the FCR in the CE group tended to increase compared with that in the CON group (Figure 1B,D). However, ADWI tended to decrease in the CE group compared with the CON group (Figure 1E). There were no statistically significant differences in ADG between the CON and the CE groups (Figure 1C). In lactating female mice, ADFI and ADG were significantly increased (*p* < 0.05) in the CE group, FCR was significantly lower in the CE group compared with the CON group (*p* < 0.05), and ADWI tended to increase (Figure 1F–I).

**Figure 2:**
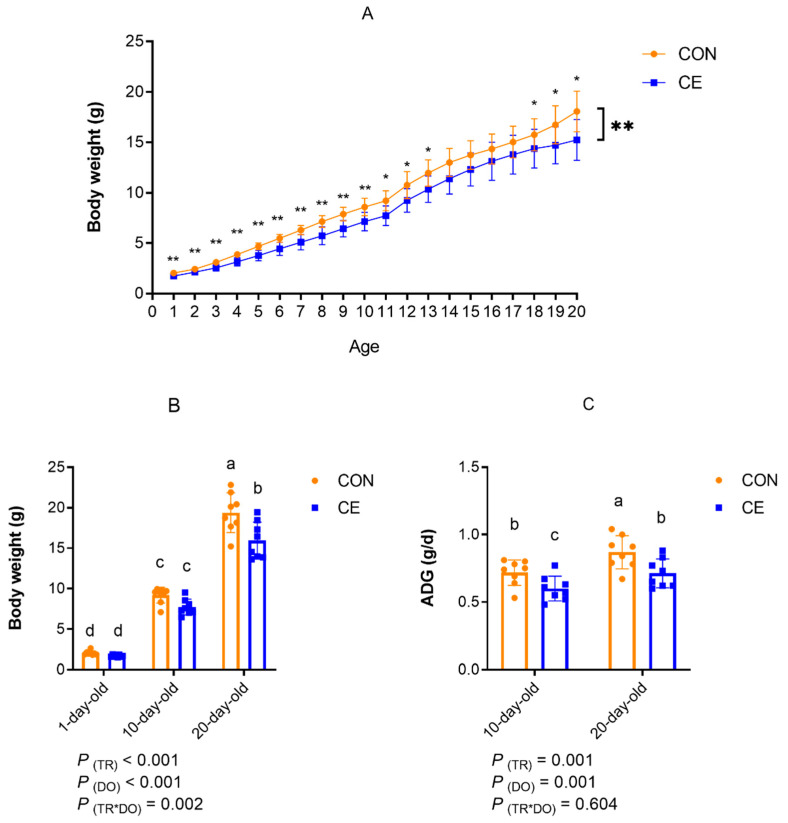
Effects of cold exposure on growth performance of mice pups. (**A**) Body weight of mice pups 1~20-days old. (**B**) Body weights of mice pups at 1-, 10-, and 20-days old. (**C**) Average daily weight gain of mice pups: ADG (average daily weight gain). Note: TR: treatment group (control group and cold exposure group); DO: days old (1-day old, 10-days old, and 20-days old). * indicates *p* < 0.05, ** indicates *p* < 0.01.Different superscript lowercase letters (a, b, c, d) denote significant differences (*p* < 0.05) for the same indicator; identical or no letter denotes no significant difference (*p* > 0.05).

The effects of cold exposure on the growth performance in mice pups are shown in Figure 2. Based on litter means, the body weight of mice pups from 1 to 20 days of birth was significantly higher in the CON group than in the CE group (*p* < 0.05) (Figure 2A). The two-factor main effect analysis of mice pup body weight results indicated that temperature was a main factor, with pups in the CE group having a significantly lower body weight than those in the CON group (*p* < 0.05). Age was also a main factor, as the body weight of pups in both the CON and CE groups significantly increased with age (*p* < 0.05). There was a significant interaction between treatment and age on the pups’ body weight (*p* < 0.05) (Figure 2B). Multiple comparisons showed that, at the same age, pups in the CE group had a significantly lower body weight than those in the CON group (*p* < 0.05). The body weights of both CON and CE group pups increased significantly with age (*p* < 0.05). The two-factor main effect analysis of ADG results shows that, with temperature as a main factor, the ADG of pups in the CE group was significantly lower than that of pups in the CON group (*p* < 0.05). Age was a main factor; the ADG of pups in both the CON and CE groups significantly increased with age (*p* < 0.05). There was no significant interaction between treatment and age on the ADG of the mice pups (Figure 2C). Multiple comparison results showed that, at the same age, the ADG of pups in the CE group was significantly lower than that of pups in the CON group, and the ADG of pups in both the CON and CE groups significantly increased with age.

### 3.2. Blood Cell Analysis

**Figure 3:**
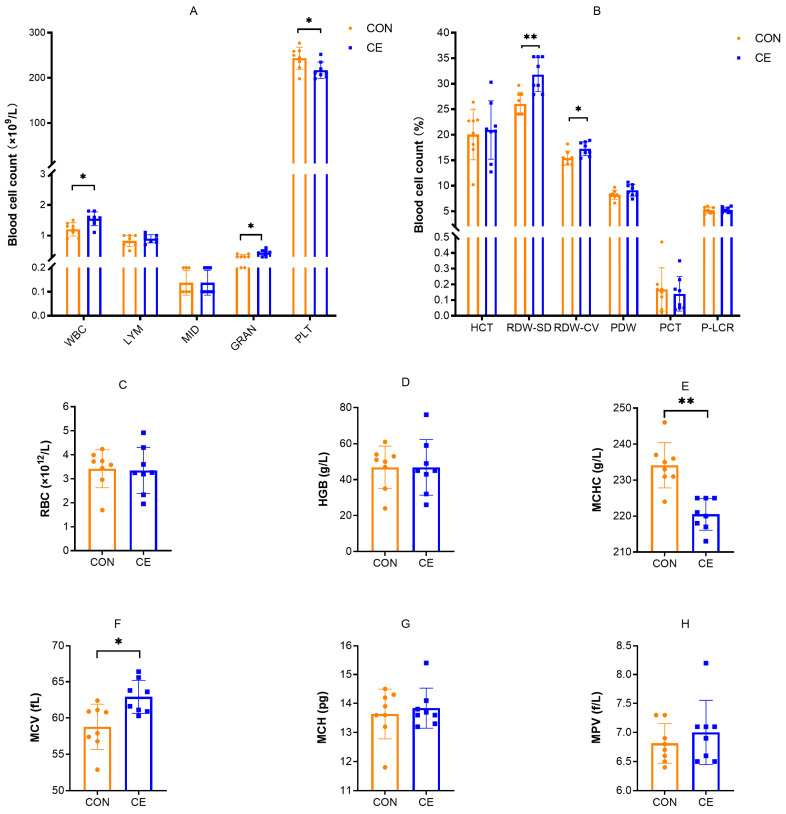
Effect of cold exposure on blood cells in female mice. (**A**) WBC (white blood cell), LYM (lymphocyte), MID (intermediate cell), GRAN (granulocyte), and PLT (platelet). (**B**) HCT (hematocrit), RDW-SD (red blood cell volume distribution width—SD), RDW-CV (red blood cell volume distribution width—CV), PDW (platelet distribution width), PCT (plateletcrit), and P-LCR (platelet larger cell ratio). (**C**) RBC (red blood cell). (**D**) HGB (hemoglobin). (**E**) MCHC (mean corpuscular hemoglobin concentration). (**F**) MCV (mean corpuscular volume). (**G**) MCH (mean corpuscular hemoglobin). (**H**) MPV (mean platelet volume). Note: * indicates *p* < 0.05, ** indicates *p* < 0.01.

The effects of cold exposure on the blood cells in female mice are presented in Figure 3. Compared to the CON group, CE female mice exhibited significant increases in WBC, GRAN, RDW-SD, RDW-CV, and MCV (*p* < 0.05) (Figure 3A,B,F). In contrast, PLT and MCHC were significantly reduced in the CE group (*p* < 0.05) (Figure 3B,G). No statistically significant differences were observed between groups for LYM, MID, HCT, PDW, PCT, P-LCR RBC, HGB, MCH, and MPV in female mice.

**Figure 4:**
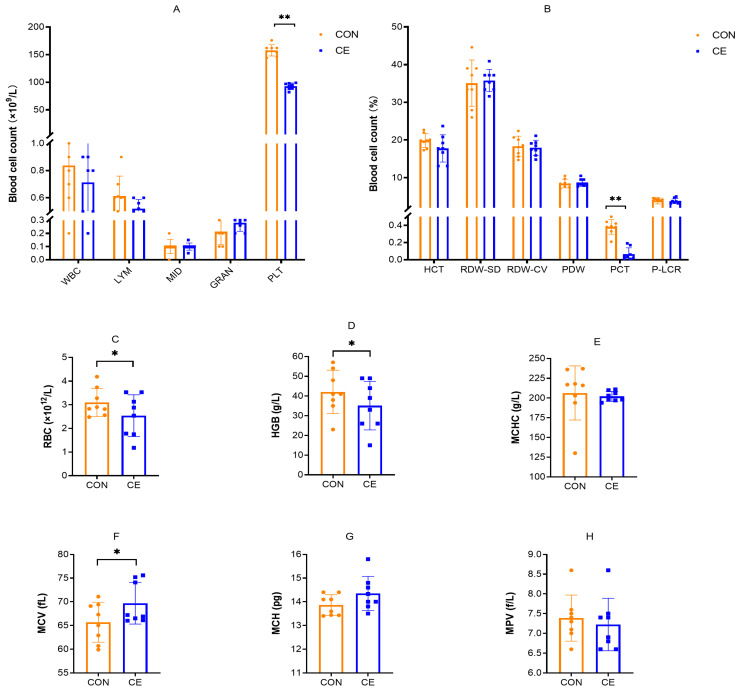
Effect of cold exposure on blood cells in 20-day-old mice pups. (**A**) WBC (white blood cell), LYM (lymphocyte), MID (intermediate cell), GRAN (granulocyte), and PLT (platelet). (**B**) HCT (hematocrit), RDW-SD (red blood cell volume distribution width—SD), RDW-CV (red blood cell volume distribution width—CV), PDW (platelet distribution width), PCT (plateletcrit), and P-LCR (platelet larger cell ratio). (**C**) RBC (red blood cell). (**D**) HGB (hemoglobin). (**E**) MCHC (mean corpuscular hemoglobin concentration). (**F**) MCV (mean corpuscular volume). (**G**) MCH (mean corpuscular hemoglobin). (**H**) MPV (mean platelet volume). Note: * indicates *p* < 0.05, ** indicates *p* < 0.01.

The effects of cold exposure on the blood cells in 20-day-old mice pups are shown in Figure 4. Compared with the CON group, CE mice pups exhibited a significant increase in MCV (*p* < 0.05) (Figure 4F). Conversely, PLT, PCT, RBC, and HGB were significantly reduced in the CE group (*p* < 0.05) (Figure 4A–D). No significant differences were observed between groups in WBC, LYM, MID, GRAN, PVC, MCH, MCHC, RDW-SD, RDW-CV, MPV, PDW, and P-LCR in mice pups.

### 3.3. Blood Biochemical and Hormone Parameters

**Figure 5:**
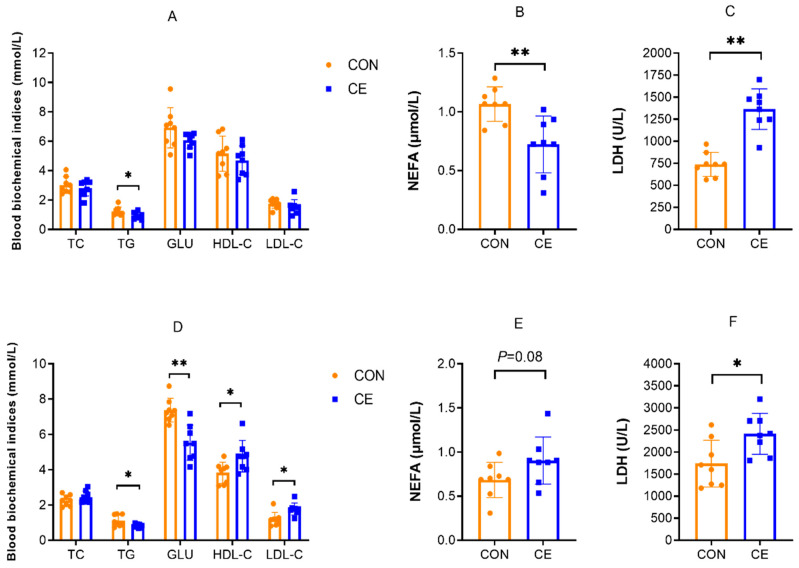
Effects of cold exposure on blood biochemical parameters. (**A**) Effects of cold exposure on blood biochemical parameters in female mice. (**B**) Effects of cold exposure on blood NEFA in female mice. (**C**) Effects of cold exposure on blood LDH in female mice. (**D**) Effects of cold exposure on blood biochemical parameters in 20-day-old mice pups. (**E**) Effects of cold exposure on blood NEFA in 20-day-old mice pups. (**F**) Effects of cold exposure on blood NLDH in 20-day-old mice pups. TC (total serum cholesterol), TG (triglyceride), GLU (glucose), HDL-C (high-density lipoprotein cholesterol), LDL-C (low-density lipoprotein cholesterol), NEFA (non-esterified fatty acids), and LDH (lactate dehydrogenase). Note: * indicates *p* < 0.05, ** indicates *p* < 0.01.

The effects of cold exposure on blood biochemical parameters are presented in Figure 5. As shown in Figure 5A–C, CE group female mice exhibited significantly higher blood LDH activity compared with the CON group (*p* < 0.05). Conversely, TG and NEFA concentrations were markedly reduced in the CE group (*p* < 0.05). No significant differences were observed in TC, GLU, HDL-C, and LDL-C concentrations between groups in female mice. In 20-day-old mice pups, the blood concentrations of HDL-C, LDL-C, and LDH were significantly higher in the CE group compared with the CON group (*p* < 0.05), while NEFA concentrations showed an increasing trend (Figure 5D–F). Notably, TG and GLU concentrations were significantly reduced in cold-exposed mice pups (*p* < 0.05) (Figure 5D). Similar to female mice, pups’ TC concentrations remained unaffected by cold exposure.

**Figure 6:**
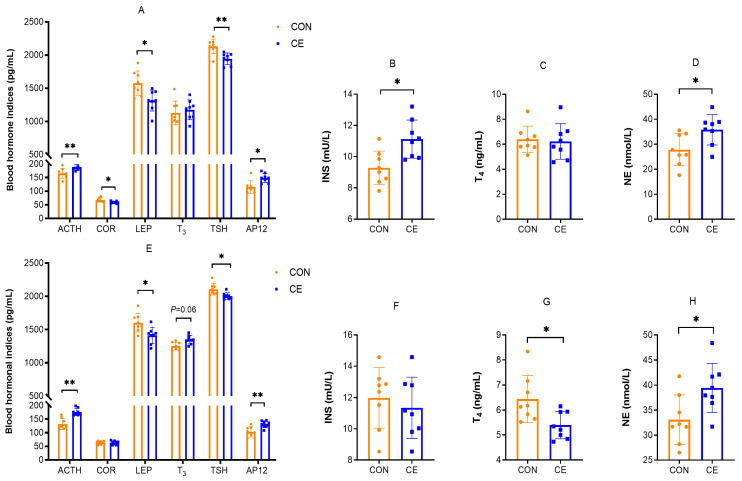
Effects of cold exposure on blood hormone parameters. (**A**) Effects of cold exposure on blood hormone parameters in female mice. (**B**) Effects of cold exposure on blood INS in female mice. (**C**) Effects of cold exposure on blood T_4_ in female mice. (**D**) Effects of cold exposure on blood NE in female mice. (**E**) Effects of cold exposure on blood hormone parameters in 20-day-old mice pups. (**F**) Effects of cold exposure on blood INS in 20-day-old mice pups. (**G**) Effects of cold exposure on blood T_4_ in 20-day-old mice pups. (**H**) Effects of cold exposure on blood NE in 20-day-old mice pups. ACTH (adrenocorticotropic hormone), COR (cortisol), LEP (leptin), T_3_ (triiodothyronine), TSH (thyroid stimulating hormone), AP12 (apelin 12), INS (insulin), T_4_ (thyroxine), and NE (noradrenaline). Note: * indicates *p* < 0.05, ** indicates *p* < 0.01.

The effects of cold exposure on hormone parameters are shown in Figure 6. In female mice, the CE group exhibited significantly elevated circulating concentrations of ACTH, AP12, INS, and NE compared with the CON group (*p* < 0.05). Conversely, COR, LEP, and TSH concentrations were significantly reduced (*p* < 0.05). No other hormonal parameters showed statistically significant alterations. (Figure 6A–D). In 20-day-old mice pups, compared with the CON group, the CE treatment group significantly increased the concentrations of ACTH, AP12 and NE (*p* < 0.05), and the T_3_ concentrations showed an increasing trend (Figure 6E,H). In contrast, TSH, LEP, and T_4_ concentrations were significantly decreased (*p* < 0.05) (Figure 6E,G). Notably, neither COR nor INS levels were significantly affected by cold exposure in 20-day-old mice pups.

### 3.4. Antioxidant Enzyme Activity

**Figure 7:**
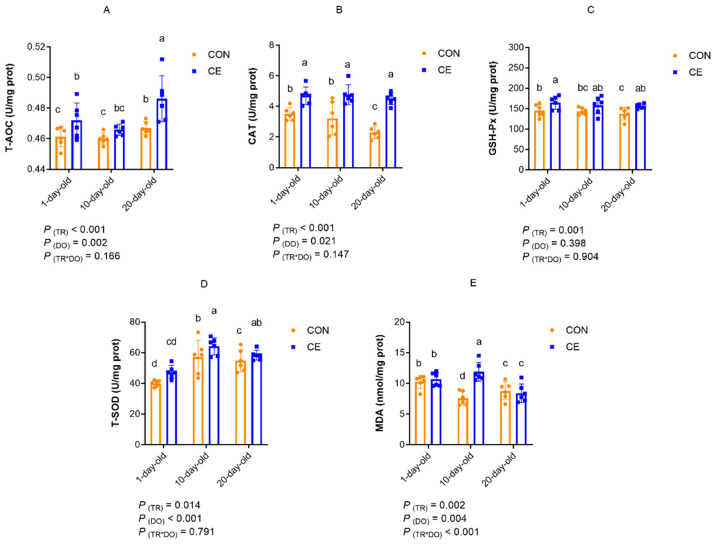
Effect of cold exposure on antioxidant enzyme activities in brown adipose tissue of mice pups. (**A**) T-AOC: total antioxidant capacity; (**B**) CAT: catalase; (**C**) GSH-PX: glutathione peroxidase; (**D**) T-SOD: total superoxide dismutase; and (**E**) MDA: malondialdehyde. Note: TR: treatment group (control group and cold exposure group); DO: days old (1-day old, 10-days old, and 20-days old). Different superscript lowercase letters (a, b, c, d) denote significant differences (*p* < 0.05) for the same indicator; identical or no letter denotes no significant difference (*p* > 0.05).

Figure 7 showed the effects of cold exposure on antioxidant enzyme activities in the BAT of mice pups. When temperature was considered as the primary factor, mice pups in the CE group exhibited significantly higher BAT T-AOC, CAT, GSH-Px, T-SOD, and MDA contents compared to the CON group (*p* < 0.05) (Figure 7A–E). With age as the main factor, the T-AOC capacity of BAT was significantly higher in pups at 20-days old than at 1- and 10-days old (*p* < 0.05) (Figure 7A). CAT activity and MDA content in BAT were significantly higher in 1- and 10-day-old mice pups than in 20-day-old mice pups (*p* < 0.05) (Figure 7B,E). The T-SOD activity in the BAT of 10- and 20-day-old mice pups was significantly higher than that of 1-day-old mice pups (*p* < 0.05) (Figure 7D). However, there was no significant difference in GSH-Px activity in the BAT of mice of different ages. There was a significant interaction between treatment and age for the levels of MDA in BAT (*p* < 0.05) (Figure 7E). There was no significant interaction between treatment and age on the capacity of T-AOC and the activities of CAT, GSH-PX, and T-SOD in BAT.

Multiple comparisons revealed that the CE treatment group significantly altered antioxidant capacity and oxidative stress markers in the BAT of neonatal mice in an age-dependent manner. In 1-day-old pups, the CE group exhibited significantly higher T-AOC and elevated the activities of CAT, GSH-Px, and T-SOD compared to the CON group (*p* < 0.05). However, MDA concentrations showed no significant difference. For 10-day-old mice pups, CAT and T-SOD activities, as well as MDA concentrations, were significantly increased in the CE group relative to CON (*p* < 0.05), whereas T-AOC and GSH-PX activity showed no significant differences. In 20-day-old mice pups, the CE treatment group significantly enhanced T-AOC, CAT, GSH-Px, and T-SOD activities again (*p* < 0.05), with no significant effect on MDA levels. In the CE treatment group, T-AOC capacity in the BAT of 20-day-old pups was higher than that of 1- and 10-day-old pups, T-SOD activity in the BAT of 10- and 20-day-old pups was higher than that of 1- and 10-day-old pups, and MDA content in the BAT of 10-day-old pups was higher than that of 1- and 20-day-old pups.

### 3.5. Relative Expression of Antioxidant mRNA

**Figure 8:**
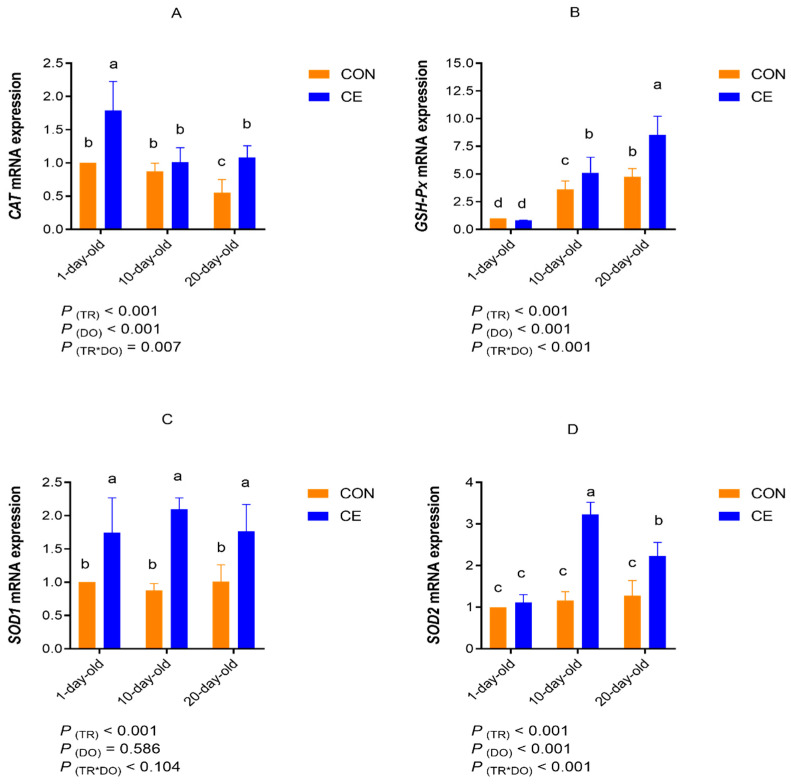
Effect of cold on antioxidant mRNA expression in brown adipose tissue of mice pups. (**A**) CAT: catalase; (**B**) GSH-Px: glutathione peroxidase; (**C**) SOD1: superoxide dismutase 1; and (**D**) SOD2: superoxide dismutase 2. Note: TR: treatment group (control group and cold exposure group); and DO: days old (1-day old, 10-days old, and 20-days old). Different superscript lowercase letters (a, b, c, d) denote significant differences (*p* < 0.05) for the same indicator; identical or no letter denotes no significant difference (*p* > 0.05).

The effect of cold exposure on the antioxidant mRNA expression in the BAT of mice pups is presented in Figure 8. According to the results of the two-factor analysis, when temperature was considered the main factor, the CE treatment group significantly increased the relative mRNA expression levels of *CAT*, *GSH-Px*, *SOD1*, and *SOD2* in the BAT higher than the CON group (*p* < 0.05). With age as a main factor, the analysis showed that the relative mRNA expression level of *GSH-Px* and *SOD2* in the BAT significantly increased with age (*p* < 0.05). However, the relative mRNA expression level of *CAT* in the BAT significantly decreased with age (*p* < 0.05). There was no significant difference in the relative mRNA expression levels of *SOD1* in the BAT across different ages. A significant interaction between temperature and age was observed for the relative mRNA expression levels of *CAT*, *GSH-Px*, and *SOD2* in the BAT of the mice pups (*p* < 0.05) (Figure 8A–D).

The results of multiple comparisons indicated that in 1-day-old mice pups, CE significantly upregulated the BAT mRNA expression of *CAT* and *SOD1* compared to the age-matched CON group. In 10-day-old mice pups, CE significantly upregulated the BAT mRNA expression of *GSH-Px*, *SOD1*, and *SOD2* compared to the age-matched CON group. In 20-day-old mice pups, CE significantly upregulated the BAT mRNA expression of *CAT*, *GSH-Px*, *SOD1*, and *SOD2* compared to the age-matched CON group. The relative mRNA expression level of *GSH-Px* in the BAT significantly increased with age in both the CON and CE groups. There was no significant difference in the relative mRNA expression level of *SOD1* across different ages within the same treatment group. The relative expression of *SOD2* mRNA was significantly upregulated in the BAT of 10-day-old pups in the CE group.

## 4. Discussion

Pregnancy is a critical biological process, essential for species survival. During early life, cold exposure is one of the survival challenges for animals. Cold exposure not only impairs growth performance but also induces direct physiological damage, such as hypothermia and frostbite. Furthermore, cold exposure can trigger systemic pathological effects, such as disruptions in energy metabolism, neuroendocrine dysfunction, impaired reproductive capacity, and cardiovascular abnormalities, which may ultimately prove fatal [28,29]. In this study, mice and their offspring were exposed to cold during gestation and lactation to systematically evaluate the effects of this exposure on growth performance and blood parameters, with a particular focus on the antioxidant capacity of BAT in the offspring.

### 4.1. Growth Performance

Cold environments adversely affect physiological functions and growth performance in animals, especially those with weaker resistance [27]. To maintain thermal homeostasis under cold exposure, two physiological strategies are typically employed: (1) increasing thermogenesis and (2) decreasing thermal dissipation [15]. In farm animals, cold exposure negatively affects growth performance and immune function, causing economic losses in animal husbandry. Cold exposure induces significant gastrointestinal responses, including accelerated gastric contraction and intestinal peristalsis, reduced intestinal chyme retention time, and increased gastric emptying rate—physiological changes that collectively stimulate appetite and enhance feed intake [30]. Furthermore, intermittent cold exposure has been associated with multiple pathological consequences in maternal physiology, such as the development of hypertension, renal dysfunction, placental abnormalities, and impaired weight gain in offspring [31]. Lian et al. [32] demonstrated that maternal body temperatures decreased under cold stress or that there was no corresponding increase in body weight despite an increase in food intake. In this present study, cold exposure increased the ADFI in pregnant female mice, but no corresponding increase in body weight was observed. Additionally, FCR showed an increasing trend, while ADWI decreased, consistent with the findings of Lian et al. [32]. These observations suggest that cold stress induced by cold exposure disrupts feed utilization efficiency in mice. One possible interpretation is that nutrients may be redirected from growth-related processes toward thermoregulatory responses; however, this interpretation is speculative, as the present study did not directly measure energy expenditure or substrate allocation. Further studies using metabolic cages or isotopic tracing are needed to clarify the underlying mechanisms. During the lactation period, cold exposure significantly increased the food intake of female mice within the first 10 days of lactation [33]. Zhao [34] also found that lactating female mice exposed to a cold environment at 5 °C consumed, on average, 2.1 g more food per day than those exposed to a warm environment at 23 °C. Similarly, the present study demonstrated that cold-exposed lactating mice exhibited elevated ADFI and ADG and reduced FCR. Female mice typically enhance food consumption to compensate for heightened energy demands, such as during lactation or cold stress [35]. These findings suggest that the increased dietary intake under cold conditions may serve two purposes: sustaining the energy requirements for thermoregulation and supporting the metabolic costs associated with milk production and lactation. Multiple prospective studies have identified significant associations between maternal exposure to adverse environmental conditions during pregnancy and behavioral abnormalities in offspring [36]. In the present study, although the body weight of pups in the CE group increased with age, it remained significantly lower than that in the CON group. Concurrently, the ADG of same-aged pups was reduced during cold exposure, aligning with the observations of Birkolo et al. [37]. These findings imply that, while maternal cold exposure did not alter offspring birth weight, it may have increased maternal energy expenditure, thereby potentially compromising fetal nutrient supply. This metabolic perturbation may have triggered adaptive responses in the pups, prioritizing cold resistance over growth and ultimately resulting in impaired physical development. It has been suggested that under cold exposure, a substantial proportion of the energy derived from maternal milk may be diverted toward maintaining homeostasis, thermoregulation, and fat deposition, leaving fewer metabolic resources available for growth in pups [4]. Consequently, the observed reduction in the pups’ ADG may be a consequence of such energy reallocation. However, given that milk composition, milk intake, and the pups’ energy expenditure were not directly measured in this study, this interpretation remains hypothetical and warrants further investigation.

### 4.2. Blood Parameters

Blood serves as a vital connective tissue and one of the most essential bodily fluids, playing a pivotal role in transporting oxygen and nutrients to tissues while simultaneously removing metabolic waste products. These functions are critical for maintaining organismal homeostasis and physiological equilibrium [38]. Blood physiological and biochemical parameters serve as reliable biomarkers, providing valuable insights into an organism’s health status and metabolic profile [39]. Routine hematological analysis enables the monitoring of alterations in blood cell counts and morphological characteristics, offering a non-invasive approach to evaluate hematological status and facilitate disease screening [40]. HGB, a specialized oxygen transport protein in erythrocytes, plays a crucial role in maintaining systemic oxygen delivery and acid-based homeostasis. Similarly, WBCs, as essential components of the immune system, perform critical functions involving pathogen defense, microbial clearance, and inflammatory response mediation. Cold exposure activates the sympathetic nervous system and triggers catecholamine release (epinephrine), which stimulates the production and mobilization of RBCs, WBCs, and PLTs [41]. In the present study, cold exposure significantly elevated WBC and GRAN counts in female mice, indicating the potential activation of immune responses under hypothermic conditions. Concurrently, we observed significant increases in MCV and red blood cell distribution width parameters (RDW-SD and RDW-CV) but a significant decrease in MCHC. An increase in the mean corpuscular volume is typically associated with enhanced oxygen-carrying capacity, as it reflects a proportional increase in hemoglobin content, but a decrease in the mean corpuscular hemoglobin concentration indicates a reduced hemoglobin concentration per cell, which may limit the overall improvement in oxygen transport [42]. This may represent an adaptive change induced by cold exposure, reflecting a compensatory regulation between oxygen transport and energy metabolism. However, its functional significance requires further verification in conjunction with tissue oxygenation indices. Such changes could represent a physiological strategy to ensure adequate oxygen supply to vital organs under cold stimulation while simultaneously supporting lactation, though direct evidence to support this interpretation is lacking. Previous studies have shown that cold exposure alters hemostatic mechanisms and increases the risk of thrombosis, with TRAP-induced platelet activation being enhanced as the temperature decreases [43]. In the present study, it was observed that platelet counts were significantly decreased in female mice exposed to cold. This change may be associated with hypothermia-induced vasoconstriction or microvascular thrombus formation, potentially reflecting increased platelet activation and consumption. Some evidence underscores the critical influence of maternal nutritional and metabolic status during key developmental windows on the offspring’s long-term health outcomes. Notably, the consequences of gestational cold exposure can extend well into adulthood [44]. This study revealed that cold exposure was associated with significantly decreased RBC counts and HGB levels in pups, along with an increase in MCV. This pattern may reflect altered erythropoiesis or accelerated erythrocyte turnover. The concomitant increase in erythrocyte volume likely serves as a compensatory mechanism to maintain oxygen delivery under cold stress [45]. Interestingly, erythrocyte distribution width parameters (RDW-SD and RDW-CV) remained unchanged in cold-exposed pups, suggesting that erythrocyte size homogeneity was preserved despite hypothermic conditions [46]. The changes in PLT and PCT observed in pups mirrored those in dams, raising the possibility of shared mechanisms, such as fetal programming or altered milk composition [47]. However, these hypotheses were not directly tested, and the precise interplay among these factors warrants further investigation.

Blood biochemical parameters are key indicators for assessing nutritional status, metabolic function, organ health, and immune responses in animals exposed to harsh environments such as cold stress [39]. LDH, a marker of liver function, is released into the bloodstream during tissue damage, making it a useful diagnostic tool for hepatic disorders [48]. Consistent with Liu et al. [49], elevated LDH activity in both females and pups was observed, suggesting the presence of cellular stress or metabolic dysfunction. LDL-C serves as the primary cholesterol transporter in the bloodstream; HDL-C is cardioprotective [50]. Studies have shown that cold exposure modulates serum HDL-C, LDL-C, and other lipid components, likely enhancing lipolysis to meet energy demands [4]. This study indicates that lipid profiles also differed between dams and pups: prolonged cold exposure decreased LDL-C levels in adult females, whereas pups exhibited increased HDL-C and LDL-C levels. This may represent differential regulatory mechanisms employed by the organism to adapt to cold exposure and fulfill energy requirements. NEFA, TG, and GLU are key interrelated components in energy metabolism that collectively regulate metabolic homeostasis. During stress responses, glucagon stimulates hepatic glycogenolysis to release GLU into circulation, thereby maintaining glucose homeostasis [51,52]. Zhou et al. [53] reported a cold stress-induced elevation of serum GLU in broiler chickens. However, this study observed reduced GLU and TG concentrations in cold exposure mice pups. This discrepancy may be attributed to species differences in thermogenic mechanisms. In broiler chickens, which lack functional BAT, cold stress primarily induces hepatic glycogenolysis, leading to elevated serum GLU levels [53]. In contrast, pups rely heavily on BAT-mediated non-shivering thermogenesis, a process that consumes substantial amounts of GLU and TG as substrates [15]. NEFA released into circulation via TG lipolysis in adipose tissue primarily function as energy substrates. Coloma-García et al. [54] reported that cold exposure stimulates NEFA mobilization as an adaptive response to meet energy demands, a process closely linked to increased NE levels and BAT activation. This study found that serum NEFA levels were reduced in lactating female mice, which may reflect the prioritization of lactation over thermoregulatory metabolism during cold exposure [55]. In pups, the elevated serum NEFA concentrations are most likely attributable to increased developmental demands, immature thermoregulatory capacity, and the activation of BAT. Cold-induced BAT activation in juveniles promotes NEFA mobilization and oxidation to support growth-related energy needs. Additionally, elevated NEFA levels in offspring may result from the intestinal absorption of NEFA and other nutrients from maternal milk [56], though this hypothesis awaits further validation. Collectively, these biochemical alterations indicate distinct metabolic adaptations to cold exposure in lactating adults versus developing offspring.

Hormones play a pivotal role in modulating physiological responses to environmental stressors such as cold exposure [57]. The hypothalamic–pituitary–adrenal (HPA) axis is a central regulator of stress responses, primarily through the secretion of ACTH and COR [56]. Cold exposure (24 h) significantly elevates plasma ACTH in mice, though findings on COR concentrations are inconsistent. Some studies reported increased COR and/or ACTH under cold exposure, while others showed a reduced or unchanged COR [58]. This discrepancy may reflect the HPA axis’ dynamic regulation, wherein moderated ACTH/COR elevation enhances gluconeogenesis and glucose/lipid mobilization to improve stress tolerance [59]. However, excessive COR may induce immunosuppression [60]. In the present study, cold exposure increased serum ACTH levels in both dams and pups, whereas COR levels were decreased in dams only. This may reflect a state of chronic stress adaptation, wherein the activated HPA axis engages negative feedback to prevent excessive stress responses. Prolonged cold exposure, particularly during lactation, likely imposes a high metabolic burden, prompting the suppression of HPA axis activity to conserve energy for thermogenesis and milk production. This interpretation is supported by elevated NE levels, which may centrally inhibit COR release, and decreased LEP levels, as LEP is known to stimulate HPA axis activity [61]. Therefore, it was hypothesized that the reduction in cortisol levels represents a physiological trade-off. Energy is prioritized for survival-critical functions over sustained stress responses under chronic cold conditions. This hypothesis requires further validation in future studies. NE, a key sympathetic neurotransmitter, regulates cardiovascular function and energy metabolism. During cold stress, NE stimulates thermogenesis via β-adrenergic receptors in BAT [62]. Accordingly, elevated serum NE levels in cold-exposed dams and pups, consistent with increased metabolic demand for thermoregulation, were observed [63]. AP12, an adipocytokine involved in glucose metabolism and insulin sensitivity [64,65], was elevated in both dams and pups under chronic cold exposure. This may represent an adaptive response to enhance energy mobilization for thermoregulation and lactation. Apelin is highly expressed in BAT, where it promotes thermogenesis and improves insulin sensitivity [66]. Insulin is a key anabolic hormone that promotes energy storage and glucose metabolism [67]. This study found elevated levels of INS in lactating female mice, which may be associated with apelin-mediated increases in insulin sensitivity and food intake, thereby promoting glucose uptake to meet energy requirements. LEP regulates energy balance by promoting appetite and suppressing thermogenesis during lactation [68]. Cold exposure significantly decreased serum LEP concentrations in both dams and pups, a finding consistent with previous reports in lactating voles [69]. This reduction may enhance thermogenesis by modulating appetite and energy expenditure, as supported by Dutra et al. [70]. Furthermore, a decrease in LEP levels may suppress the secretion of TSH and thyroid hormones [61]. Thyroid hormones, primarily T_4_ and its bioactive form T_3_, play crucial roles in thermoregulation. Studies have shown that T_3_ enhances thermogenesis by increasing UCP1 expression in brown adipocytes and elevating tissue metabolic rate [4,71]. In this study, chronic cold exposure reduced serum T_4_ levels in pups and TSH levels in both dams and pups. It was speculated that these changes may reflect enhanced BAT activity and increased local T_4_-to-T_3_ conversion, which could conserve energy for growth by downregulating thyroid axis activity [72]. Thus, the observed decrease in TSH and thyroid hormone concentrations likely represents an adaptive energy-saving mechanism in response to chronic cold exposure [73], but this mechanism requires further investigation.

### 4.3. Antioxidant Function of Brown Adipose Tissue

Cold exposure, as a typical environmental stressor, initially triggers systemic physiological changes reflected by alterations in growth performance and blood parameters. Specifically, cold exposure induces changes in systemic energy substrates, including decreased GLU and TG levels and increased NEFA concentrations in offspring, indicating elevated metabolic demand and enhanced substrate mobilization to cope with the cold stress. However, this adaptive process promotes the production of endogenous ROS. Concurrently, NE levels rise in the blood of both dams and offsprings. NE is known not only to trigger BAT thermogenesis via β-adrenergic signaling but also to serve as a potential upstream signal for activating antioxidant defenses [54,74]. The onset and progression of oxidative stress within BAT can directly impair its thermogenic function and metabolic efficiency, thereby disrupting systemic energy balance. This disruption ultimately manifests as changes in blood parameters (energy metabolism-related and stress-related biochemical indicators) and growth performance [75]. Conversely, blood parameters can objectively reflect the systemic stress level and metabolic status resulting from an impaired BAT antioxidant capacity or disrupted ROS clearance, serving as macro-level biological indicators of tissue functional status. Therefore, investigating whether chronic cold exposure induces adaptive antioxidant responses in BAT is of critical importance.

Extreme temperatures disrupt redox homeostasis and induce oxidative stress, thereby impairing organismal health and development [27]. This stress causes damage to critical biomolecules, including membrane lipids, proteins, and nucleic acids through ROS-mediated oxidation [74]. During cold stress, elevated metabolic activity increases mitochondrial ROS production [76]. When ROS generation exceeds the capacity of clearance mechanisms, systemic oxidative damage occurs, potentially contributing to neurodegenerative and inflammatory pathologies [77]. The antioxidant defense system plays a crucial role in protecting against oxidative stress, with its enzymatic components primarily comprising CAT, SOD, and GSH-Px. SOD catalyzes the dismutation of superoxide anion radicals (•O_2_^−^) into the less reactive H_2_O_2_, which is subsequently broken down into water and oxygen by CAT and GSH-Px [78]. T-AOC provides a comprehensive assessment of both enzymatic (SOD, CAT, and GSH-Px) and non-enzymatic (ascorbic acid and α-tocopherol) antioxidant activity [79]. As the end product of lipid peroxidation, MDA serves as a reliable biomarker of oxidative damage. The ROS-initiated peroxidation of membrane unsaturated fatty acids generates MDA, making it a sensitive indicator of membrane oxidative damage [80]. Thus, MDA levels are frequently used as an important marker for evaluating the extent of oxidative damage.

Cold exposure potently activates uncoupled oxidative phosphorylation in BAT and alters redox homeostasis by modulating key antioxidants such as MDA, GSH, and CAT. The antioxidant defense system in BAT relies on the coordinated expression of CAT, SOD2, and GPx to scavenge ROS and mitigate oxidative damage [81]. Consistent with Đurić et al. [79], who reported that chronic cold exposure increases interscapular BAT mass and elevates the GSH content and antioxidant enzyme activities in mice, this study found that cold exposure significantly enhanced antioxidant enzyme activities, T-AOC, and the mRNA expression of antioxidant enzymes in mice pups. These findings indicate that cold exposure activates the antioxidant defense system in BAT, enhancing both the uncoupling and antioxidant capacity in response to cold stress. However, the concurrent rise in MDA levels suggests excessive ROS production, likely driven by BAT thermogenesis (UCP1-mediated mitochondrial electron leakage) [82]. Although compensatory antioxidant mechanisms are activated, the elevated MDA indicates that oxidative damage may temporarily dominate. Whether BAT can maintain long-term protection through dynamic equilibrium remains to be determined. Our findings also reveal age-dependent antioxidant responses in BAT. Studies have shown that cold exposure-induced mitochondrial oxidative stress in BAT elevates superoxide production and oxidative damage markers, while simultaneously triggering age-dependent enhancement of antioxidant defenses in developing pups [83]. The findings in this study demonstrated that, although BAT activity declined with age, cold exposure in 10- and 20-day-old pups exhibited significantly higher T-SOD activity and T-AOC than newborns, with 20-day-old pups showing peak antioxidant capacity and reduced MDA content. This enhanced protection correlates with the upregulated mRNA expression of antioxidant enzymes, indicating a developmental transition toward more robust BAT antioxidant systems during chronic cold adaptation. These tissue-specific responses contrast with previous reports of cold-induced oxidative damage in other organs [84]. While Lubkowska et al. [84] observed decreased SOD, CAT, and GSH-Px activities in rat erythrocytes, livers, kidneys, and hearts during cold exposure, this study demonstrated the significant enhancement of these antioxidant enzymes and T-AOC in mice pups’ BAT, consistent with previous findings in rats [85]. This discrepancy likely reflects BAT’s unique tissue properties. Cold exposure may also activate the Nrf2-Keap1 pathway in BAT to preserve redox balance under increased metabolic demand [86]; however, this mechanism was not directly assessed in the present study and warrants further investigation. Collectively, these findings suggest that cold exposure activates the antioxidant function of BAT in developing mice pups, which mitigates oxidative damage through specific compensatory mechanisms and plays a crucial role in defending against oxidative stress.

This study has several limitations that should be acknowledged when interpreting the results. First, direct evidence linking BAT antioxidant adaptation to thermogenicity is lacking; therefore, functional thermogenic assays are required to validate its proposed role in cold‑induced thermogenesis. Second, the interpretation of endocrine regulatory mechanisms remains largely inferential and requires mechanistic confirmation via future studies on receptor expression and intracellular signaling pathways. Third, this study was conducted exclusively in mice under a fixed cold exposure protocol, limiting the generalizability of its findings to other animal models, livestock species, or varying cold intensities and durations. Future research should integrate direct functional measurements, causal interventions (agonist/antagonist administration, gene knockout, or tissue-specific manipulation), and cross-species comparisons to validate the regulatory networks linking endocrine adaptation, BAT antioxidant function, and thermogenesis under cold exposure.

## 5. Conclusions

This study provides an integrated assessment of physiological responses to chronic cold exposure in dams and offspring, with a focus on tissue-specific antioxidant adaptation in the BAT of pups. Cold exposure increased ADFI and decreased FCR in female mice during gestation and lactation, while impairing growth in their offspring. Dams and pups exhibited distinct alterations in blood parameters, indicating different adaptive strategies to cope with cold stress. Notably, cold exposure significantly activated the antioxidant capacity of BAT in developing pups, as evidenced by increased T-AOC, antioxidant enzyme activities, and corresponding gene expression. This BAT-specific antioxidant adaptation contributes to mitigating cold-induced oxidative damage during early development. Collectively, these findings provide preliminary evidence for the coordinated regulation of endocrine function and redox homeostasis under cold exposure.

## Figures and Tables

**Table 1:** Formula and nutrient levels of the basal diet.

Items	Content (%)
Corn	40.00
Soybean meal	15.50
Fish meal	4.00
Wheat flours	18.00
Wheat bran	18.00
Salt	0.50
CaHPO_4_	2.00
Limestone	1.00
Premix ^1^	1.00
Total	100.00
Nutrient levels ^2^	
Crude protein	18.00
EE	4.00
CF	5.00
Ash	8.00
Lys	0.82
Met + Cys	0.53
Ca	1.00
Phosphorus	0.60

^1^ Main Components of Premix: Na 2 g/kg, K 5 g/kg, Mg 2 g/kg, Cu 10 mg/kg, Fe 100 mg/kg, Zn 30 mg/kg, Mn 75 mg/kg, I 0.5 mg/kg, Se 0.1 mg/kg, Vitamin A 7000 IU/kg, Vitamin D 800 IU/kg, Vitamin E 60 IU/kg, Vitamin K 3 mg/kg, Vitamin B1 8 mg/kg, Vitamin B2 10 mg/kg, Vitamin B6 6 mg/kg, Vitamin B12 0.022 mg/kg, Biotin 0.1 mg/kg, Niacin 45 mg/kg, Pantothenic Acid 17 mg/kg, Folic Acid 4 mg/kg, and Choline 1250 mg/kg. ^2^ Nutrient levels are all calculated values.

## Data Availability

The original contributions presented in this study are included in the article. Further inquiries can be directed to the corresponding author.

## Abbreviations

The following abbreviations are used in this manuscript:

BAT	brown adipose tissue
MCV	mean corpuscular volume
PLT	platelet count
LDH	lactate dehydrogenase
NEFA	non-esterified fatty acid
ACTH	adrenocorticotropic hormone
AP12	apelin 12
INS	insulin
NE	norepinephrine
COR	cortisol
Lep	leptin
TSH	thyroid-stimulating hormone
PCT	plateletcrit
RBC	red blood cells
HGB	hemoglobin
T_4_	thyroxine
T-AOC	total antioxidant capacity
CAT	activities of catalase
GSH-Px	glutathione peroxidase
T-SOD	superoxide dismutase

## Appendix A

**Table A1:** Primer sequences used for the study.

Primer Name	Gene Back NO.	Primer Sequence (5′-3′)	Fragment Size (bp)
*CAT*	NM_009804.2	F: AGGCTCAGCTGACACAGTTC	223
		R: ATGGAGAGACTCGGGACGAA	
*GSH-Px*	NM_008160.6	F: CCACGTGATCTCAGCACCAT	114
		R: AGAAGGCATACACGGTGGAC	
*SOD1*	NM_011434.2	F: GTCGGCTTCTCGTCTTGCTC	80
		R: CTGATGGACGTGGAACCCAT	
*SOD2*	NM_013671.3	F: GCCCAAACCTATCGTGTCCA	70
		R: AGGGAACCCTAAATGCTGCC	
*GAPDH*	NM_001289726.2	F: AAGAGGGATGCTGCCCTTAC	119
		R: TACGGCCAAATCCGTTCACA	

Note: CAT (catalase), GSH-Px (glutathione peroxidase), SOD1 (superoxide dismutase 1), SOD2 (superoxide dismutase 2), and GAPDH (Glyceraldehyde-3-Phosphate Dehydrogenase).
